# Commentary: The Modulation of Chaihu Shugan Formula on Microbiota Composition in the Simulator of the Human Intestinal Microbial Ecosystem Technology Platform and Its Influence on Gut Barrier and Intestinal Immunity in Caco-2/THP1-Blue™ Cell Co-Culture Model

**DOI:** 10.3389/fphar.2022.904890

**Published:** 2022-06-01

**Authors:** Yang Yu, Cun Liu, Jingyang Liu, Jing Zhuang, Changgang Sun

**Affiliations:** ^1^ College of Traditional Chinese Medicine, Shandong University of Traditional Chinese Medicine, Jinan, China; ^2^ College of Traditional Chinese Medicine, Weifang Medical University, Weifang, China; ^3^ College of First Clinical Medicine, Shandong University of Traditional Chinese Medicine, Jinan, China; ^4^ Department of Oncology, Weifang Traditional Chinese Hospital, Weifang, China

**Keywords:** chaihu shugan formula, simulator of the human intestinal microbial ecosystem (SHIME), gut microbiota, gut barrier, intestinal immunity

In a recent issue of this journal, we read with great interest the experimental report by Yao and coworkers (Liu et al., 2022), focusing on the application of the Simulator of the Human Intestinal Microbial Ecosystem (SHIME) to study the traditional Chinese medicine (TCM)—Chaihu Shugan Formula (CSF) and its metabolites on the modulation of gut microenvironment: gut microbiota, gut barrier, and intestinal immunity. This study sheds light on the positive impact of CSF on gut health, and provides a new perspective for exploring the mechanism of the TCM compound on the intestinal microbial ecosystem.

Intestinal microecology has been a research hotspot in the field of gut-associated diseases, especially digestive system tumors. Intestinal dysbiosis is the main cause of the formation of hypoxia, low pH, inflammatory and even interstitial high pressure environment, which promotes the formation of tumor microenvironment and provides conditions for tumor generation, progression and metastasis (Sepich-Poore et al., 2021; Park et al., 2022).

In intestinal flora research, the integration between *in vitro* and *in vivo* studies is complementary and necessary. However, it must be mentioned that human studies related to the gut microbiota are often limited. In addition to collecting feces, sampling of small intestine, ascending colon, transverse colon and other parts is limited for normal people. Based on the difficult sample collection process and the tremendous inter-individual variability between samples caused by confounders such as genetic background, diet, health status, lifestyle and environmental conditions (Sauvaitre et al., 2021), as an alternative, researchers have developed several intestinal bioreactors to simulate and study the metabolic fate of food, microorganisms and generating bioactive compounds (microbial metabolites) in the digestive system for several weeks, including TNO gastro-Intestinal Model (i.e., TIM-1 for the stomach and small intestinal model and TIM-2 for the colon system) (Minekus et al., 1999), Reading model (Gibson et al., 1988), Simulator of the Human Intestinal Microbial Ecosystem (SHIME) model (Molly et al., 1993), PolyFermS model (Dostal et al., 2013; Fehlbaum et al., 2015), etc. Among them, SHIME as a multi-stage sequential batch model of semi-continuous culture is a unique scientifically validated *in vitro* alternative to the whole gastrointestinal tract. The SHIME system consists of five reactors, which sequentially simulate three regions of the stomach (acidic conditions and pepsin digestion), small intestine (digestion process) and large intestine, namely the ascending, transverse, and descending portions of the colon. The first two reactors use fill and draw principles to simulate the different steps of food intake and digestion. The peristaltic pump allows the addition of defined amounts of SHIME nutrient medium, pancreatic enzymes and bile in the small intestine to achieve gastric content transfer. Retention time and the pH of different vessels are carefully controlled to obtain complex and stable microbial communities that are highly similar to those in different regions of the human gastrointestinal tract in structure and function.

However, it must be pointed out that, like any other *in vitro* model, the SHIME system has certain limitations. First, the SHIME system suffers from the lack of the physiological environment and host responses, such as the absence of oxygen gradients that is released from the blood to the mucus layer in the body. Furthermore, movement and mixing along the gastrointestinal tract is carried out by pumping and stirring rather than by peristalsis (Falduto et al., 2022). Meanwhile, the model uses porcine gastric mucin with lower molar mass and more impurities than freshly prepared gastric mucin (Pham and Mohajeri, 2018), and the absorption of water and microbial metabolites is not routinely simulated in the colonic compartment. Despite these shortcomings, the SHIME model allows the status of various regions of the gastrointestinal tract to be studied independent from host context.

In summary, we completely agree that Yao et al. used the SHIME reactor device to study the important effects of TCM compounds on gut microbial ecology. These data innovatively transforms the research of complex TCM ingredients into the representative of intestinal flora, and are the creative experimental results of using the SHIME system to study the regulation of human intestinal microbiota by TCM compounds. This innovative study mediated by the SHIME model provides a new understanding of the mechanism of TCM compound intervention in the intestinal microenvironment, which is an interesting, encouraging attempt to promote multidisciplinary integration.
